# Vulvar Lymphangiectasia After Therapy for Cervical Cancer: A Case Report and Literature Review

**DOI:** 10.3390/jcm14051675

**Published:** 2025-03-01

**Authors:** Vincenzo Pinto, Christopher Clark, Doriana Di Nanni, Amerigo Vitagliano, Grazia Pinto, Gerardo Cazzato

**Affiliations:** 1 Unit of Obstetrics and Gynecology, Department of Interdisciplinary Medicine, University of Bari, 70124 Bari, Italy; vincenzo.pinto@uniba.it (V.P.); christopher.clark@uniba.it (C.C.); amerigo.vitagliano@uniba.it (A.V.); 2 Section of Molecular Pathology, Department of Precision and Regenerative Medicine and Ionian Area (DiMePRe-J), University of Bari “Aldo Moro”, 70124 Bari, Italy; doriana.dinanni@policlinico.ba.it; 3 Dentistry Unit, Department of Interdisciplinary Medicine, University of Bari Medical School, 70124 Bari, Italy; grazia.pinto@uniba.it

**Keywords:** vulvar lymphangiectasia, lymphatic obstruction, histopathology, conservative management

## Abstract

**Background**: Vulvar lymphangiectasia (VLA) is a rare condition characterized by the abnormal dilation of lymphatic vessels in the vulvar region, often secondary to surgery or radiation therapy for malignancies. Its clinical presentation closely resembles other dermatological conditions, posing challenges for accurate diagnosis and appropriate management. This study aims to present a rare case of VLA occurring decades after cervical carcinoma surgery, contributing to the limited literature on this condition and offering insights into its differential diagnosis and management. **Methods:** A 70-year-old female patient presented with multiple fluid-filled vesicles in the vulvar region appearing 36 years after undergoing radical hysterectomy with pelvic lymphadenectomy for cervical carcinoma. The lesions were biopsied, and histopathological and immunohistochemical analyses were performed to confirm the diagnosis. A review of the existing literature on VLA was conducted to contextualize this case. **Results:** A histopathological examination revealed papillomatous lesions with hyper-keratosis, dilated lymphatic vessels, and no signs of atypia, consistent with VLA. An immunohistochemical analysis confirmed the lymphatic nature of the lesions. Due to the patient’s comorbidities, asymptomatic presentation, and lesion stability, conservative management with regular follow-up was chosen. No progression or complications were observed during the 12-month follow-up period. **Conclusions**: This case highlights the importance of considering VLA in patients presenting with vulvar vesicles, especially those with a history of lymphatic disruption. An accurate diagnosis through histopathological and immunohistochemical techniques is essential to distinguish VLA from other conditions. Conservative management may be appropriate for asymptomatic cases, but tailored therapeutic strategies are needed to address symptomatic or disfiguring lesions.

## 1. Introduction

The term lymphangiectasia (LA) refers to an abnormal dilation of deep lymphatic vessels, which normally occurs due to obstructions in lymphatic flow and is often associated with lymphedema. This condition must not be confused with lymphangioma, which refers to an abnormal growth of lymphatic vessels due to epithelial and stromal proliferation [1] occurring at birth or in early childhood, usually before the age of 5. Therefore, while the former is considered the result of increased pressure inside lymph vessels and is an acquired condition which arises from multiple possible causes, the latter is better defined as a benign tumor and has been conventionally divided into classical lymphangioma circumscriptum (LC) and localized lesions [2]. However, the clinical and histological findings of LA and congenital LC are very similar. 

Although the exact pathogenesis is not clear, LA is thought to arise from damage to previously normal deep lymph vessels, which alters lymph flow and determines an increase in local hydrostatic pressure, causing the saccular dilation of lymph vessels and vesicle formation [3].

Lymphangiectasia is a rare condition, which has been mainly described as a result of surgical or radiological treatment of malignancies, such as breast cancer [4]. Other causes, although less common, include scleroderma, Crohn’s disease, and infectious diseases, such as tuberculosis or filariasis, which increase the hydrostatic pressure of lymphatic vessels through scar tissue formation or the direct obstruction of lymph flow [5].

An even rarer localization of LA is the vulva. Most cases reported in the literature describe acquired forms of vulvar lymphangiectasia (VLA) secondary to tubercular lymphadenitis [6], Crohn’s disease [7], genital mutilation, or previous surgery and/or radiation treatment for underlying malignancies, such as cervical carcinoma [8,9,10,11,12]. Given the rarity of this condition and the ambiguity of its clinical presentation, the risks of misdiagnosis and mistreatment are elevated [13]. Indeed, vulvar LA is frequently mistaken for other much more common lesions, usually vulvar condylomas [14].

In this paper, we present a case of VLA, describing the patient’s past medical history and clinical presentation and including pathological findings in the hope of aiding other clinicians in diagnosing this rare but challenging disease.

We also considered the differential diagnosis between other causes of vulvar skin vesicles and reviewed the literature surrounding this topic.

## 2. Case Presentation

A 70-year-old female patient was referred to our institution with a history of pedunculated vesicles on her vulvar area, which appeared after surgery for cervical cancer. She reported that the nodules had slightly increased both in number and size over the past 2 years, although their size seemed to have stabilized in the 2 months preceding our observation. The patient did not complain of any subjective symptoms, such as pain, burning, or itching.

**Figure 1:**
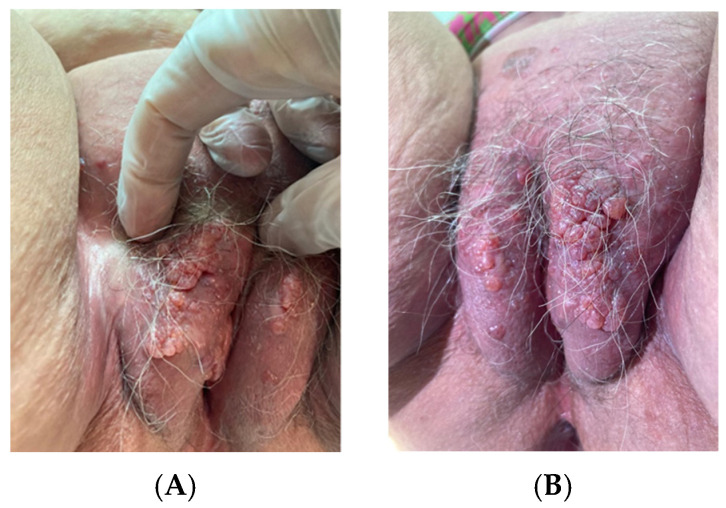
(**A**) Lateral view showing vesicles 3–12 mm in diameter, mainly on the left labium majus with a “cluster-like” organization (**B**).

On examination, the patient showed multiple pinkish fluid-filled vesicles with a smooth surface scattered bilaterally over the labia majora and extending to the left inguinocrural fold. The vesicles were 3–12 mm in diameter and were more evident at the level of the left labium majus, where they showed a “cluster-like” organization (Figure 1A,B). No fluid discharge from the vesicles nor signs suggestive of superadded infections were detected. The lesions did not evidence the typical “frog-spawn” appearance of lymphangioma circumscriptum. The labia minora were normal considering the patient’s age. The inguinal lymph nodes were not enlarged. An obvious lymphoedema was not evident at the external genitalia nor at the lower limbs.

The patient’s past medical history is significant for Wertheim’s hysterectomy with pelvic lymphadenectomy for cervical carcinoma which the patient underwent 36 years prior. No radiotherapy was associated with the surgical treatment of the disease. Follow-up examinations were always normal.

Biopsy specimens were obtained by isolating whole vesicles with electrosurgery microneedle set on cutting mode.

**Figure 2:**
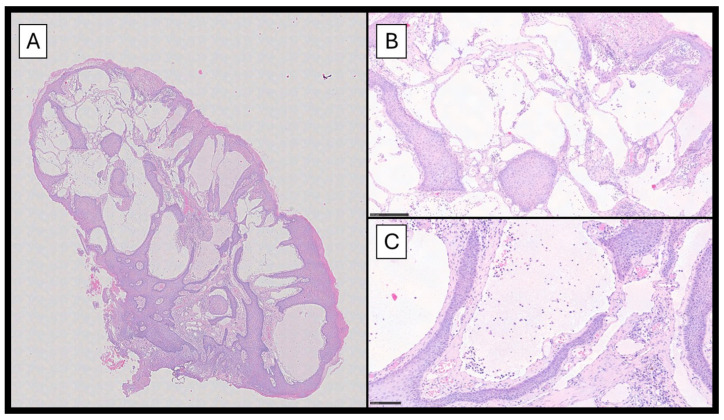
(**A**) A histopathological photomicrograph showing a papillomatous lesion characterized by hyperkeratosis, focal erosion, and multiple dilated vessels (hematoxylin–eosin, original magnification 4×). (**B**) An image with higher magnification showing details of the dilated lymphatic vessels lined by flat endothelium containing some lymphocytes and serum (**C**) (hematoxylin–eosin, original magnification 10× and 20×).

**Figure 3:**
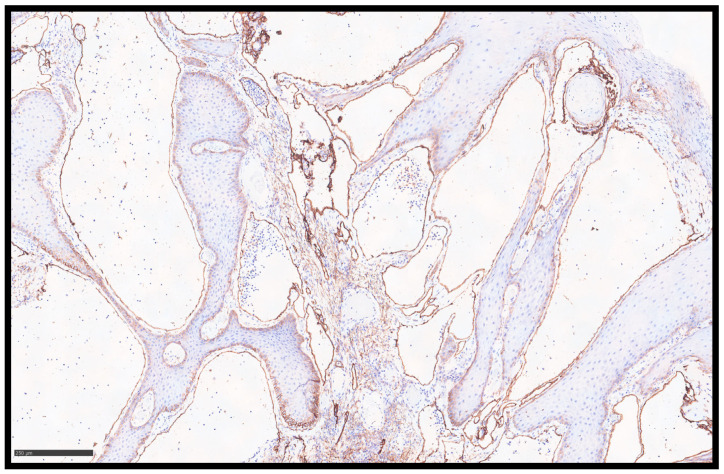
An immunohistochemical investigation with anti-D2-40 antibody showing the endothelial layer of the lymphatic vessels (immunohistochemistry, original magnification 10×).

Histopathological examination showed a papillomatous lesion characterized by hyperkeratosis, focal erosion, and dilated vessels lined by flat endothelial cells without atypia containing lymphocytes and serum (Figure 2A–C); furthermore, the lymphatic nature of the vessels was confirmed by immunohistochemical reaction with Podoplanin (D2-40) (Figure 3).

Surgical treatment was not taken into consideration due to the patient’s comorbidities (i.e., diabetes mellitus and obesity), the absence of symptoms, and the stability of the lesions. Therefore, we decided to follow up with the patient at regular, short-term appointments, and the lesions did not show an increase in size 6 and 12 months from diagnosis.

## 3. Discussion

Lymphatic drainage from the vulva is supplied by a network of lymphatic vessels in the internal and external superficial area of the labia minora and majora, which runs to the lymphatic collectors, continuing to the mons pubis and towards the superficial inguinal nodes. These lymph nodes are also connected to the deep inguinal nodes through efferent lymphatic trunks [15]. Any condition altering the anatomical structure of lymph nodes and/or the lymphatic trunks connecting these nodal stations could theoretically cause a condition of LA. Therefore, the pathogenesis of acquired LA is believed to be an isolated but often deep abnormality of the lymphatics, as demonstrated by lymphangiograms which have shown the dilated lymphatics to be totally separate from the main drainage apparatus [16].

The most evident clinical manifestation of LA is the formation of vesicles, generally appearing in the form of clusters rather than diffused all over the area. These lesions may evolve from vesicles to papules or nodules as per tissue organization, thus rendering clinical diagnosis even more challenging [17]. The color of the vesicles varies from normal pinkish to red or purple according to the presence and quantity of blood inside the lymph vessels, which can be found due to the frequent hemangiolymphatic connections [18]. Occasionally, fluid-filled vesicles can rupture and produce chronic fluid discharge; moreover, ruptured vesicles may act as entry points for infection, especially for common cutaneous saprophytes, and ultimately result in chronic or recurrent cellulitis [18]. In some cases, ineffective lymphatic drainage may lead to lymphedema of the whole vulva or of the legs. The typical “frog-spawn vesicles” sign which should prompt the diagnosis is rarely found and probably more indicative of the primary lesion, lymphangioma circumscriptum. Vesicles closely resemble other more common dermatological conditions, and differential diagnosis with neoplasms, condylomas, herpes genitalis, or molluscum contagiosum may be challenging. In particular, the skin overlying the vesicles may be thickened, hyperkeratotic, or clearly verrucous, and this may be the reason why these lesions are so commonly mistaken for acuminate condylomas. Multiple vulvar fibroepithelial polyps are the lesions that mimic VLA vesicles the most, but they are also frequently found in the vagina [19]; in any case, a histopathological examination is always decisive.

In further detail regarding the histopathological view, a retrospective examination covering 15 years at King Edward Memorial Hospital in Perth, Western Australia, described eight cases of VLA in patients with documented iatrogenic disruption of pelvic lymphatic drainage [20]. Clinical data, including the patient’s age, lesion distribution, lymphoedema presence, and initial presumptive diagnoses, were extracted from pathology request forms and medical records. Interestingly, in several instances, the preliminary clinical assessment suggested lymphangioma circumscriptum or vulval edema; however, some cases indicated the possibility of viral infection. The histopathological assessment of the lesions demonstrated a range of epidermal alterations from mild acanthosis to prominent pseudoepitheliomatous hyperplasia; the epidermis frequently exhibited hyperkeratosis and parakeratosis, characterized by an uneven extension of rete ridges, with occasional findings of focal papillomatous alterations. Apart from sporadic nuclear atypia and keratinocyte hypertrophy, conclusive indicators of HPV infection, including koilocytosis, were lacking. 

The most notable histological observation was the appearance of significantly dilated lymphatic vessels in the superficial layers. These channels, occasionally displaying valve-like intraluminal folds, were regarded as the diagnostic feature of VLA. An immunohistochemical analysis elucidated the diagnosis with the endothelial cells of the dilated vessels that consistently expressed CD31 and D2-40 while exhibiting a notable absence of CD34 expression—a characteristic that distinguishes VLA from other vascular lesions such as angiokeratoma, which generally maintain CD34 positivity. Supplementary immunostaining for p16 yielded negative results, further excluding HPV-associated lesions, while p53 expression was seen only focally [20]. All of these data confirm the diagnosis in our case.

In a recent paper, some authors conducted a retrospective analysis of cases from their institution alongside a systematic literature study [21]. Through a natural language search utilizing keywords such as “vulva lymphangioma”, “lymphatic vulva”, “lymphangiectasia vulva”, and “malformation vulva” in their pathology information system, they discovered instances of vulvar lymphangioma circumscriptum that were preceded by a malignancy diagnosis. Cases related to non-neoplastic diseases, such as Crohn’s disease or infections, were eliminated to focus exclusively on the malignancy-associated group. The diagnosis was confirmed through the examination of glass slides by two board-certified dermatopathologists and the use of immunohistochemistry staining for D2-40 to confirm lymphatic endothelial cells. Extensive clinical data were collected, and the parameters covered the patient’s age at malignancy and VLA diagnosis, malignancy type, the period between the two diagnoses, and details of prior treatment, including radiation therapy and lymph node dissection. The evaluation of 71 cases—including 65 sourced from the PubMed database across 25 publications spanning from 1977 to 2016 and 6 cases from their own institution—revealed a median age of 39.5 years at malignancy diagnosis and a median age of 54.5 years at VLA diagnosis. The median duration from the onset of the primary malignancy to the emergence of VLA was 10 years, with a broad range of 0 to 32 years. A significant majority (91.4%) of these patients had received radiation therapy as part of their cancer treatment, and around 70.2% had undergone lymph node dissection. The primary antecedent malignancy was cervical carcinoma, comprising 71.8% of cases, with cervical squamous cell carcinoma being identified as the most prevalent subtype upon further classification. Additional cancers comprised vulvar carcinoma, endometrial carcinoma, Hodgkin lymphoma, rhabdomyosarcoma, rectal carcinoma, melanoma, and vaginal carcinoma.

Clinically, VLA manifested with multiple lesions that were characterized by an exophytic vesicular or verrucous appearance, and they may have presented as transparent, fluid-filled vesicles ranging from 1 to 5 mm in size or as merging papules and nodules exhibiting a smooth, flesh-toned surface. In many instances, the lesions had a pseudo-verrucous or papillomatous morphology. Alongside the local manifestation, concomitant symptoms were prevalent. Almost fifty percent of the patients exhibited simultaneous lymphedema in the lower limbs. Additional reported symptoms encompassed lymphatic drainage (in almost one-third of patients), pain, recurring bouts of cellulitis or erysipelas, hemorrhage, itching, and psychosocial distress. Notwithstanding the clinical significance of these findings, fewer than fifty percent of the cases presented an adequate clinical differential diagnosis that encompassed lymphangioma. In some cases, the lesions were erroneously identified as condyloma acuminatum (genital warts), molluscum contagiosum, herpes infection, or angiokeratoma or were merely referred to in vague terminology such as “lesion” or “lump”.

The microscopic analysis of the lesions demonstrated uniform characteristics. All instances exhibited cystically dilated lymphatic channels, regarded as the hallmark of VLA. These channels were generally located directly beneath the epidermis; however, in certain cases, they penetrated into the mid-to-deep dermis. The superficial epidermis frequently displayed papillomatous or pseudo-verrucous alterations accompanied with acanthosis, hyperkeratosis, parakeratosis, and, in certain regions, erosion. Immunohistochemical labeling utilizing D2-40 proved to be an effective diagnostic method as it specifically marked the lymphatic endothelial cells that border the dilated channels. In several instances, there were localized radiation-induced alterations in the surrounding tissue, encompassing cutaneous fibrosis and the formation of atypical fibroblasts. Nonetheless, these alterations were typically constrained, and their interpretation was hampered by the intrinsic challenge of differentiating minor radiation effects from modifications directly caused by the lymphangioma. The authors highlight the difficulty of accurately diagnosing VLA. Due to its clinical rarity and diverse manifestations, misdiagnosis frequently occurs. Pathologic misinterpretations have included diagnoses such as low-grade squamous intraepithelial lesions and angiokeratoma, and the diagnostic challenges are exacerbated by the occurrence of VLA, which frequently manifests years after the original cancer treatment, thus dissociating the lesion from its etiological setting in clinical history.

This article additionally addresses the overarching nomenclature employed for these lesions. Despite the suggestion of words like “lymphatic malformation” and “lymphangiectasia” to more accurately represent the non-neoplastic characteristics of the condition, the authors opted for “lymphangioma circumscriptum” to maintain consistency with previous research in this field. They emphasize the necessity of acknowledging that AVLC, despite its innocuous histological appearance, can have substantial clinical ramifications, particularly when linked to the aftermath of prior cancer therapies [21].

As already mentioned, in the literature, VLA has been found to appear after radical surgery followed by radiotherapy for cervical cancer [8,9,10,12,14,18]. After cervical cancer therapy, obesity is considered the most dominant additional risk factor for VLA development [14]. The mean interval between cervical cancer therapy and the development of VLA was 17 years (range 7–31 years) [22]. Hong reported a case of VLA after surgery and radiotherapy which occurred 35 years after cervical cancer management [10]. In our case, VLA appeared 36 years after surgery. It occurred after radical hysterectomy and lymphadenectomy alone because the patient did not undergo radiotherapy. Some authors, on the contrary, believe that radiotherapy is a major contributor to pathogenesis due to the architectural disruption or secondary fibrosis of lymphatic vessels at the base of the reticular dermis and at the fat–reticular dermis junction [18]. The stenosis of dermal lymphatics results in increased pressure up-stream and leads to saccular dilation of the superficial lymphatic channels with the formation of vesicles [18]. Although many cases of VLA available in the literature have been diagnosed in women of Asian ethnicity, evidence suggests that no racial predominance is found in this disease [23]. A recent systematic review of the topic was inconclusive on this subject due to the lack of ethnicity data included in the study [11].

The treatment of vulvar lymphangiectasia has not been standardized yet. The most common therapeutic options reported in the literature vary from surgical excision to ablative techniques (CO_2_ laser vaporization and electrocoagulation). Laser vaporization is a frequently reported treatment, which also proved to be a safe method in patients not eligible for surgery, although the recurrence rates appear to be higher [24]. Among the less conventional treatment options, the combination of vesicle radiofrequency ablation with a fine-needle electrode followed by sclerotherapy using 3% intralesional polidocanol in the subcutaneous tissue has been proposed [9]. Some authors have investigated the feasibility of lymphaticovenous anastomosis to reduce the recurrence rate of this disease in women with proven communication between the vulva and the lymphatic vessels in the lower limb with encouraging results [25]. Other authors suggest performing extended resection and subsequent pudendal flap reconstruction to treat very large lesions [12]. In asymptomatic cases with non-disfiguring lesions, observation following tissue sampling may also be considered.

A high recurrence rate after LA therapy is reported and is directly proportional to the original lesion size [26]. Due to the rarity of vulvar cases, it is not simple to report the recurrence rate after therapy. However, the recurrence rate reported after surgery for all cases of lymphangioma circumscriptum (primary and acquired) is 23% during follow-up periods ranging from 6 to 81 months [12,26]. In a small series of women with VLA treated with a wide local excision, Yoon reported a similar recurrence rate of about 25% [22].

Cases resulting in malignancy are exceptionally rare, and those which have been reported in the literature are correlated with the congenital lymphangioma circumscriptum condition and not to LA (acquired lymphangioma).

Based on the high recurrence rate and on the available literature, treatment strategies need to be carefully tailored by taking different variables into account, such as the referred symptoms, patient’s comorbidities, and anatomical features. Conducting a follow-up of asymptomatic lesions is a therapeutic option.

## Data Availability

Data is contained within the article.
